# The influence of the administration sequence of neoadjuvant immunotherapy combined with chemotherapy on the postoperative pathological response of stage IIA-IIIB non-small cell lung cancer

**DOI:** 10.1186/s12885-026-16028-9

**Published:** 2026-04-22

**Authors:** Yu Zhang, Zixin Li, Aiju Zeng, Daiyuan Ma

**Affiliations:** Department of Oncology, Affiliated Hospital of North Sichuan Medical College, No. 1, Maoyuan South Road, Shunqing District, Nanchong, Sichuan 637000 China

**Keywords:** Non-small cell lung cancer, Neoadjuvant immunotherapy, Administration sequence, Pathological response, Propensity score matching

## Abstract

**Background:**

This study aimed to evaluate whether the within-cycle sequence of neoadjuvant immunochemotherapy influences postoperative pathological response in patients with stage IIA–IIIB non-small cell lung cancer (NSCLC).

**Methods:**

We retrospectively enrolled 192 patients with stage IIA–IIIB NSCLC who received neoadjuvant immunochemotherapy. Patients were divided into Group A (chemotherapy on day 1 followed by immunotherapy on day 2, *n* = 122) and Group B (immunotherapy on day 1 followed by chemotherapy on day 2, *n* = 70). Propensity score matching (1:1, caliper = 0.2) created 62 matched pairs. The primary endpoint was postoperative pathological response including pathological complete response and major pathological response. Conditional logistic regression was used, and E‑values were calculated for sensitivity analysis. Secondary endpoints included objective response rate (ORR), safety, and perioperative outcomes.

**Results:**

After matching, baseline characteristics were well balanced. The pathological response rate was higher in Group A than in Group B (50.00% vs. 30.64%; univariate OR = 2.33, 95%CI 1.07–5.09, *P* = 0.033; multivariable OR = 4.78, 95%CI 1.24–18.46, *P* = 0.023). E‑value analysis suggested that the observed association may be relatively robust to unmeasured confounding (univariate E = 4.09, multivariable E = 9.03). ORR was also higher in Group A (72.58% vs. 51.65%; OR = 2.62, 95%CI 1.16–5.93, *P* = 0.020). Grade ≥ 3 treatment‑related adverse events were similar between groups (35.48% vs. 29.03%, *P* = 0.494). Operative time was longer in Group A (180 vs. 165 min, *P* = 0.037), but no differences were observed in R0 resection rate, postoperative complications, or other surgical outcomes.

**Conclusion:**

In patients with stage IIA–IIIB NSCLC, the within-cycle administration sequence of chemotherapy followed by immunotherapy was associated with a higher postoperative pathological response rate. This dosing sequence may have clinical optimization value, but further validation through prospective studies is still needed.

**Supplementary Information:**

The online version contains supplementary material available at 10.1186/s12885-026-16028-9.

## Introduction

Lung cancer remains the leading cause of cancer‑related morbidity and mortality worldwide, with non‑small cell lung cancer (NSCLC) accounting for approximately 82% of all cases [1, 2]. Although surgery is the cornerstone of curative treatment for early‑stage NSCLC, about 30%–55% of patients experience postoperative recurrence, metastasis, or death [3, 4]. Effective perioperative systemic therapy is therefore essential to eliminate micrometastases, reduce relapse, and improve long‑term survival [5].

Neoadjuvant therapy offers the advantage of treating the tumor within an intact immune microenvironment. It can downstage disease, convert initially unresectable tumors to resectable ones, and increase the R0 resection rate [6, 7]. Standard neoadjuvant treatment was historically platinum‑based doublet chemotherapy [8], which yielded modest improvement in progression‑free survival (PFS) and a very low pathological complete response (pCR) rate (< 5%) [8, 9]. The emergence of immune checkpoint inhibitors (ICIs), particularly programmed death receptor-1 (PD-1)/ programmed death receptor-ligand 1 (PD-L1) inhibitors, has revolutionized perioperative management of NSCLC. By blocking PD-1 / PD-L1 axis, these agents reinvigorate antitumor immunity [10, 11]. In the neoadjuvant setting, they may exploit high tumor antigen load and existing immune infiltration, generating robust systemic responses and immunological memory [12, 13]. The pivotal phase III CheckMate 816 trial demonstrated that adding nivolumab to neoadjuvant chemotherapy significantly improved pCR (24.0% vs. 2.2%) and event‑free survival (EFS) in resectable stage IB–IIIA NSCLC [12]. Based on these results, major guidelines now recommend neoadjuvant immunochemotherapy as a standard option [14, 15].

Pathological response depth correlates strongly with long‑term outcomes. pCR and major pathological response (MPR) are well‑validated surrogate endpoints [16]. A meta‑analysis indicated approximately 70% 5‑year survival and 20% recurrence in patients achieving pCR after neoadjuvant immunochemotherapy [17]. Moreover, patients who achieve pCR may safely omit adjuvant immunotherapy [18]. Thus, optimizing neoadjuvant strategies to maximize pathological response is critical for improving survival.

Despite the established efficacy of neoadjuvant immunochemotherapy, a fundamental practical detail – the sequence of chemotherapy and ICI administration within a treatment cycle – remains unstandardized and its biological impact largely overlooked. Most clinical protocols and practices administer both agents on the same day, ignoring potential sequence‑dependent effects on the tumor microenvironment. Emerging evidence from other tumors suggests sequence matters. In locally advanced esophageal squamous cell carcinoma, toripalimab given 48 h after chemotherapy achieved a higher pCR rate than concurrent administration (36.4% vs. 7.7%) [19]. A preclinical study in melanoma, lung cancer, and peritoneal cancer models showed that chemotherapy‑first schedules produced superior tumor control and survival compared with immunotherapy‑first or concurrent regimens, attributed to increased intratumoral effector T cells and reduced immunosuppressive cells [20]. Conversely, in BRAF wild‑type metastatic melanoma, ipilimumab before chemotherapy yielded better outcomes, possibly by expanding tumor‑specific T‑cell clones before chemotherapy‑induced lymphodepletion [21]. Another trial in advanced pancreatic cancer found no survival advantage with a chemotherapy‑first sequence [22]. These conflicting data underscore that the optimal sequence may be tumor-specific.

In NSCLC, while neoadjuvant immunochemotherapy has become standard, the optimal administration sequence within a cycle has not been established [12]. We therefore conducted a retrospective study directly comparing two sequences. It was hypothesized that the chemotherapy-first sequence, by leveraging immunogenic cell death (ICD) and remodeling the tumor microenvironment, might be associated with deeper pathological responses. These findings may help refine the clinical execution of neoadjuvant immunochemotherapy.

## Patients and methods

### Study design and participants

This single‑center retrospective cohort study consecutively enrolled patients with stage IIA–IIIB NSCLC who were evaluated by a multidisciplinary team and scheduled for radical surgery at the Affiliated Hospital of North Sichuan Medical College between January 1, 2020, and June 30, 2025. The study was approved by the hospital’s ethics committee (approval No. 2025ER197-1).

#### Inclusion criteria

1. Histologically confirmed NSCLC, staged IIA–IIIB according to the 9th edition of the IASLC TNM classification [23].
2. Eastern Cooperative Oncology Group performance status 0–1.
3. Confirmed absence of EGFR, ALK, and ROS1 mutations.
4. First‑line neoadjuvant treatment with a PD‑1/PD‑L1 inhibitor combined with platinum‑doublet chemotherapy.
5. 2–4 cycles of neoadjuvant therapy.
6. Consistent treatment regimen and sequence in all cycles.
7. Complete clinical data.

#### Exclusion criteria

1. Contraindications to surgery at diagnosis.
2. Intolerance to chemotherapy or immunotherapy.
3. Prior malignancy or antitumor therapy.
4. Use of radiotherapy or anti‑angiogenic agents during neoadjuvant or perioperative period.
5. Coexisting hematologic, autoimmune, or systemic diseases requiring long‑term immunosuppressants or glucocorticoids.
6. Severe cardiac, cerebral, renal, or hepatic dysfunction.

#### Group definition

Based on the actual clinical workflow at our center, patients received chemotherapy and immunotherapy on consecutive days to improve tolerability and manage fluid load. The two groups were defined as follows: Group A (chemotherapy‑first): Chemotherapy on day 1 followed by immunotherapy on day 2; Group B (immunotherapy‑first): Immunotherapy on day 1 followed by chemotherapy on day 2. They had the same overnight interval. All patients received treatment in 3‑week cycles. The sequence was verified through electronic medical orders and nursing records. Once the treatment decision by a multidisciplinary team (MDT) was made, patients were referred to the oncology chemotherapy center and managed by a dedicated attending physician. The treatment regimen followed established guidelines. However, because the optimal administration sequence remained undefined, which is precisely the question we aimed to investigate, the sequence was determined based on the attending physician’s routine practice and preference.

### Data collection

#### Pathological diagnosis

Tumor specimens were obtained by bronchoscopy or computerized tomography (CT)‑guided percutaneous biopsy. Diagnosis and subtyping were confirmed independently by two senior pathologists, using hematoxylin‑eosin (HE) staining and immunohistochemistry.

#### Clinical staging

Baseline clinical stage was determined by enhanced chest CT, and N status was confirmed by endobronchial ultrasound‑guided transbronchial needle aspiration or mediastinoscopy. Distant metastasis was excluded by abdominal ultrasound, brain Magnetic Resonance Imaging or CT, bone scan, and in some cases, Positron Emission Tomography-CT was acceptable.

#### Neoadjuvant treatment regimens

All patients received 2–4 cycles of a PD‑1/PD‑L1 inhibitor (sintilimab, tislelizumab, or camrelizumab, all at a fixed dose of 200 mg) plus platinum‑doublet chemotherapy. For non‑squamous NSCLC: pemetrexed 500 mg/m² and cisplatin 75 mg/m² or carboplatin area under the curve (AUC) 5. For squamous NSCLC: paclitaxel 175 mg/m² and cisplatin 75 mg/m² or carboplatin AUC 5. Chemotherapy was administered on day 1 and immunotherapy on day 2 for Group A, and the reverse for Group B, as described above.

#### Imaging evaluation

CT scans were performed at baseline and 4 weeks after completion of neoadjuvant therapy. Objective response was assessed according to Response Evaluation Criteria in Solid Tumors version 1.1 (RECIST v1.1) [24]. Objective response rate (ORR) was defined as the proportion of patients achieving complete or partial response sustained for ≥ 4 weeks.

#### Surgery

After the MDT discussion, patients who were deemed suitable for surgery were randomly assigned to the thoracic surgeons. Surgery was scheduled 4–6 weeks after the last treatment cycle, provided no disease progression or intolerable adverse events occurred. The surgical approach (video‑assisted thoracoscopic surgery vs. thoracotomy) was at the discretion of the attending surgeon. Standard procedures included complete tumor resection and systematic ipsilateral hilar and mediastinal lymph node dissection. Resection status was classified as R0 (microscopically margin‑negative), R1 (microscopically positive), or R2 (gross residual). Perioperative data, including operative time, blood loss, length of hospital stay, and postoperative complications, were recorded.

#### Postoperative pathological evaluation

Surgical specimens were assessed by at least two experienced pathologists according to International Association for the Study of Lung Cancer recommendations [25]. The primary endpoint was postoperative pathological response, defined as the sum of pathological complete response (pCR: no viable tumor cells in the primary tumor or lymph nodes) and major pathological response (MPR: ≤10% residual viable tumor cells in the primary tumor, regardless of nodal status). Waterfall plots were constructed to visualize the degree of tumor regression.

To initially explore potential histological differences, three pairs of HE‑stained slides (six patients total) of patients who achieved pCR were randomly selected. Two pathologists, blinded to group assignment, assessed the following features: (1) Immune cell infiltration: Lymphocyte density, tertiary lymphoid structures; (2) Stromal reaction: Necrosis, fibrosis, granulomas; (3) Tumor bed characteristics: Foam cells, cholesterol clefts. Disagreements were resolved by consensus. These observations are hypothesis‑generating and do not provide definitive mechanistic evidence.

#### Safety assessment

Adverse events during neoadjuvant therapy were graded using Common Terminology Criteria for Adverse Events Version 5.0 (CTCAE v5.0) [26]. Grade ≥ 3 treatment‑related adverse events (TRAEs) were recorded, including hematologic toxicity, liver/kidney dysfunction, and immune‑related events. Severe adverse outcomes including death or Intensive Care Unit (ICU) admission were also noted. Postoperative complications within 30 days were collected.

### Statistical analysis

All analyses were performed using R 4.5.1. Two‑tailed *P* < 0.05 was considered statistically significant.

To mitigate selection bias in this retrospective study, propensity score matching (PSM) was employed. A logistic regression model including age, sex, smoking history, histology, tumor location, clinical T stage, clinical N stage, immunotherapy agent, and number of cycles was used to estimate propensity scores. Patients from Group A and Group B were matched 1:1 using nearest‑neighbor matching with a caliper of 0.2 times the standard deviation of the logit of the propensity score. Standardized mean differences (SMD) were used to assess balance. SMD < 0.1 indicated good balance and was considered as qualified.

For the primary endpoint (pathological response) and secondary endpoints (ORR and TRAEs), all matched patients (including those who did not undergo surgery) were included in the analysis. Non‑surgical patients were classified as not having achieved pathological response. Conditional logistic regression was performed on the matched pairs. Variables with SMD > 0.1 after matching were included in multivariable models to adjust for residual confounding [27, 28]. Results are reported as odds ratios (OR) with 95% confidence intervals (CI). E‑values were calculated to evaluate the robustness of the main findings to unmeasured confounding. The E‑value represents the minimum strength of association that an unmeasured confounder would need to have with both treatment and outcome to fully explain the observed effect. Larger E‑values indicate greater robustness [29].

For perioperative outcomes, analysis was performed on the subgroup of patients who actually underwent surgery. Baseline balance was re‑assessed in this subgroup. Continuous variables were compared using the independent‑samples t‑test or Mann‑Whitney U test as appropriate; categorical variables were compared using the chi‑square test or Fisher’s exact test.

## Results

### Baseline characteristics

#### Propensity score matching

**Fig. 1:**
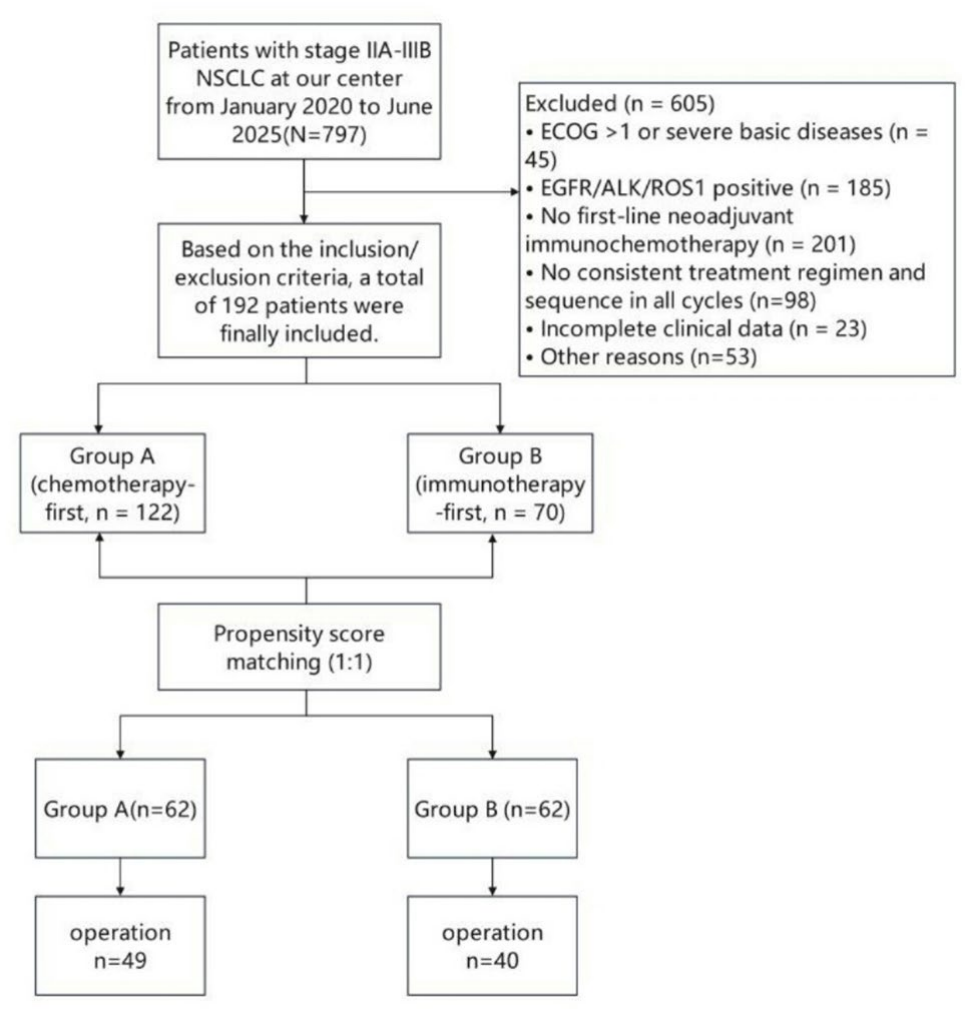
Patient flow diagram

Figure 1 shows the patient flow diagram. A total of 192 patients met the inclusion criteria, comprising 122 in Group A (chemotherapy‑first) and 70 in Group B (immunotherapy‑first). Before PSM, there were imbalances in T stage and immunotherapy agent between groups. After 1:1 PSM, 62 matched pairs were successfully created. All baseline variables were well balanced after matching, with all SMD < 0.2 (Table 1). After PSM, the distribution of platinum agents was well balanced between groups. Cisplatin was used in 28 patients (45.2%) in Group A and 26 patients (41.9%) in Group B, while carboplatin was used in 34 patients (54.8%) and 36 patients (58.1%), respectively. There was no significant difference between groups (*P* = 0.716).

**Table 1:** Baseline characteristics before and after propensity score matching

Characteristic	Before PSM					After PSM				
	All (*N* = 192)	Group A (*n* = 122, %)	Group B (*n* = 70, %)	*P*	SMD	All (*N* = 124)	Group A (*n* = 62, %)	Group B (*n* = 62, %)	*P*	SMD
Gender				0.524					1	
Male	178 (92.71)	112 (91.80)	66 (94.29)		-0.09	117 (94.35)	59 (95.16)	58 (93.55)		0.075
Female	14 (7.29)	10 (8.20)	4 (5.71)		0.09	7 (5.65)	3 (4.84)	4 (6.45)		-0.075
Age (year)				0.891					0.281	
≥ 65	100 (52.08)	64 (52.46)	36 (51.43)		0.021	60 (48.39)	27 (43.55)	33 (53.23)		-0.195
<65	92 (47.92)	58 (47.54)	34 (48.57)		-0.021	64 (51.61)	35 (56.45)	29 (46.77)		0.195
Smoking				0.584					0.488	
Yes	158 (82.29)	99 (81.15)	59 (84.29)		-0.08	101 (81.45)	49 (79.03)	52 (83.87)		-0.119
No	34 (17.71)	23 (18.85)	11 (15.71)		0.08	23 (18.55)	13 (20.97)	10 (16.13)		0.119
Histology				0.819					0.783	
Squamous	166 (86.46)	106 (86.89)	60 (85.71)		0.035	109 (87.9)	55 (88.71)	54 (87.10)		0.051
Adenocarcinoma	26 (13.54)	16 (13.11)	10 (14.29)		-0.035	15 (12.1)	7 (11.29)	8 (12.90)		-0.051
Tumor location				0.531					0.857	
Left lung	104 (54.17)	64 (52.46)	40 (57.14)		-0.094	65 (52.42)	32 (51.61)	33 (53.23)		-0.032
Right lung	88 (45.83)	58 (47.54)	30 (42.86)		0.094	59 (47.58)	30 (48.39)	29 (46.77)		0.032
T stage				**0.021**					0.873	
1	29 (15.1)	15 (12.30)	14 (20.00)		-0.235	18 (14.52)	10 (16.13)	8 (12.90)		0.088
2	62 (32.29)	37 (30.33)	25 (35.71)		-0.117	43 (34.68)	20 (32.26)	23 (37.10)		-0.104
3	59 (30.73)	35 (28.69)	24 (34.29)		-0.124	47 (37.9)	23 (37.10)	24 (38.71)		-0.033
4	42 (21.88)	35 (28.69)	7 (10.00)		0.413	16 (12.9)	9 (14.52)	7 (11.29)		0.092
N stage				0.500					0.883	
0	49 (25.52)	35 (28.69)	14 (20.00)		0.206	30 (24.19)	16 (25.81)	14 (22.58)		0.116
1	35 (18.23)	22 (18.03)	13 (18.57 )		0.008	20 (16.13)	9 (14.52)	11 (17.74)		-0.055
2	108 (56.25)	65 (53.28)	43(61.43)		-0.102	74 (59.68)	37 (59.68)	37 (59.68)		-0.096
Immunotherapy agent				**< 0.001**					0.678	
Sintilimab	89 (46.35)	43 (35.25)	46 (65.71)		-0.638	74 (59.68)	35 (56.45)	39 (62.90)		-0.09
Tislelizumab	55 (28.65)	38 (31.15)	17 (24.29)		0.148	33 (26.61)	17 (27.42)	16 (25.81)		0.036
Camrelizumab	48 (25)	41 (33.61)	7 (10.00)		0.500	17 (13.71)	10 (16.13)	7 (11.29)		0.092
Treatment cycle				0.388					0.907	
2	63 (32.81)	36 (29.51)	27 (38.57)		-0.199	42 (33.87)	20 (32.26)	22 (35.48)		-0.069
3	65 (33.85)	42 (34.43)	23 (32.86)		0.033	42 (33.87)	21 (33.87)	21 (33.87)		0
4	64 (33.33)	44 (36.07)	20 (28.57)		0.156	40 (32.26)	21 (33.87)	19 (30.65)		0.068

Data are presented as n (%). P values < 0.05 are shown in bold

*SMD* Standardized mean difference, *PSM* Propensity score matching

### Clinical outcomes before PSM

Before PSM, the postoperative pathological response rate was 47.54% (58/122) in Group A and 31.43% (22/70) in Group B (OR = 1.98, 95%CI 1.07–3.65, *P* = 0.024). The ORR was 68.03% (83/122) in Group A and 52.86% (37/70) in Group B (OR = 1.90, 95%CI 1.04–3.46, *P* = 0.058). The incidence of grade ≥ 3 TRAEs was 27.87% (34/122) in Group A and 30% (21/70) in Group B (OR = 0.90, 95%CI 0.47–1.72, *P* = 0.752).

### Surgical eligibility and reasons for non-surgery after PSM

Of the 124 matched patients, 89 (71.77%) underwent radical surgery, 49/62 (79.03%) in Group A and 40/62 (64.52%) in Group B. The surgery rate did not differ significantly between groups (OR = 2.12, 95%CI 0.92–4.92, *P* = 0.079). Reasons for not undergoing surgery are detailed in Table 2.

**Table 2:** Reasons for not undergoing surgery after matching

Reason	Group A (*n* = 13)	Group B (*n* = 22)
Progression/ in efficacy	4	6
Treatment toxicity	3	5
Patient refusal	2	4
Unresectable at surgery	2	4
Other adverse events	2	3

### Perioperative outcomes after PSM

Baseline characteristics of the surgical subgroup remained well balanced (Table 3). Perioperative outcomes are summarized in Table 4. Operative time was significantly longer in Group A (median 180 min vs. 165 min, *P* = 0.037), but there were no differences in the proportion of video‑assisted thoracoscopic surgery, unplanned conversions to thoracotomy, lobectomy, the number of lymph node dissections, the R0 resection rate, the blood loss volume, the length of hospital stay, postoperative complications, and the 30-day mortality rate/ICU admission rate. Postoperative complications were graded according to the Clavien–Dindo classification (Table 5).

**Table 3:** Baseline characteristics of the surgical subgroup

Characteristic	Total (*n* = 89)	Group A (*n* = 49)	Group B (*n* = 40)	*P*	SMD
Sex				1.000	
Male	84 (94.38)	46 (93.9)	38 (95.0)		-0.047
Female	5 (5.62)	3 (6.12)	2 (5.00)		0.047
Age				0.386	
≥ 65 years	40 (44.94)	20 (40.82)	20 (50.00)		-0.187
< 65 years	49 (55.06)	29 (59.18)	20 (50.00)		0.187
Smoking history				0.864	
Yes	75 (84.27)	41 (83.67)	34 (85.00)		-0.036
No	14 (15.73)	8 (16.33)	6 (15.00)		0.036
Histology				0.806	
Squamous	77 (86.52)	42 (85.71)	35 (87.50)		-0.051
Adenocarcinoma	12 (13.48)	7 (14.29)	5 (12.50)		0.051
Tumor location				0.602	
Left lung	45 (50.56)	26 (53.06)	19 (47.50)		0.111
Right lung	44 (49.44)	23 (46.94)	21 (52.50)		-0.111
T stage				0.852	
1	16 (17.98)	10 (20.41)	6 (15.00)		0.134
2	31 (34.83)	17 (34.69)	14 (35.00)		-0.006
3	32 (36.96)	16 (32.65)	16 (40.00)		-0.157
4	10 (11.24)	6 (12.24)	4 (10.00)		0.068
N stage				0.710	
0	27(30.34)	16 (32.65)	11 (27.50)		0.190
1	13 (14.61)	7 (14.29)	6 (15.00)		0.089
2	49 (55.06)	26 (53.06)	23 57.50)		-0.111
Immunotherapy				0.818	
Sintilimab	52 (58.43)	28 (57.14)	24 (60.00)		-0.058
Tislelizumab	25 (28.09)	15 (30.61)	10 (25.00)		0.122
Camrelizumab	12 (13.48)	6 (12.24)	6 (15.00)		-0.084
Treatment cycles				0.522	
2	37 (41.57)	18 (36.73)	19 (47.50)		-0.133
3	30 (33.71)	17 (34.69)	13 (32.50)		0.046
4	22 (24.72)	14 (28.57)	8 (20.00)		0.160

**Table 4:** Perioperative outcomes in the surgical subgroup

Outcome	Group A (*n* = 49)	Group B (*n* = 40)	OR (95% CI)	P值
VATS, n (%)	40 (81.63)	33 (82.50)	1.06 (0.36,3.15)	0.916#
Unplanned conversion to thoracotomy, n (%)	4 (8.16%)	2 (5.00)	-	0.680*
Lobectomy, n (%)	38 (77.55)	34 (85.00)	0.61 (0.20,1.81)	0.370#
lymph nodes harvested [number, median (IQR)]	15 (12–18)	14 (11–18)	-	0.623†
R0 resection, n (%)	47 (95.92)	35 (87.50)	-	0.236*
Blood loss [mL, median (IQR)]	100 (100,130)	100 (100,157.5)	-	0.086†
Operative time [min, median (IQR)]	180 (150,240)	165(147.5,182.5)	-	0.037†
Postoperative stay [days, median (IQR)]	6 (5, 8)	6 (4, 9.25)	-	0.612†
Postoperative complications, n (%)	10 (20.41)	11 (27.50)	0.68 (0.25,1.80)	0.430#
30-day death/ICU admission, n (%)	4 (8.16)	3 (7.50)	-	1.000*

*Abbreviations*: *VATS* Video‑assisted thoracoscopic surgery, *IQR* Interquartile range, *ICU* Intensive care unit

#Chi‑square test; *Fisher’s exact test; †Mann‑Whitney U test

**Table 5:** Postoperative complications by Clavien–Dindo grade

Clavien – Dindo grade	Complication	Group A (*n* = 10)	Group B (*n* = 11)
I	Pneumothorax	2	1
	Pleural effusion	1	0
	Hemothorax	0	1
II	Pneumothorax	1	1
	Arrhythmia	1	0
	Infection	0	1
III	Pneumothorax	0	1
	Bronchopleural fistula	0	1
	Pleural effusion	1	1
	Hemothorax	1	1
IV	Respiratory failure	0	1
	Infection	1	1
V	Respiratory failure	1	0
	Infection	1	1

### Clinical outcomes

#### Postoperative pathological response

The primary endpoint was analyzed in all 124 matched patients, including those who did not undergo surgery. 31 patients (50%) in Group A achieved postoperative pathological response (11 MPR and 20 pCR), compared with 19 patients (30.64%) in Group B (5 MPR and 14 pCR). Conditional logistic regression showed a significantly higher response rate in Group A (univariate OR = 2.33, 95%CI 1.07–5.09, *P* = 0.033). After adjusting for variables with SMD > 0.1 after matching (age, smoking, T stage, N stage), the association remained significant (multivariable OR = 4.78, 95%CI 1.24–18.46, *P* = 0.023) (Table 6).

**Table 6:** Conditional logistic regression for postoperative pathological response

Variable	Univariate analysis		Multivariable analysis	
	*P*	OR (95%CI)	*P*	OR (95%CI)
Group				
Group A		1.00 (Reference)		1.00 (Reference)
Group B	**0.033**	2.33 (1.07–5.09)	**0.023**	4.78 (1.24–18.46)
Age (year)				
≥ 65		1.00 (Reference)		1.00 (Reference)
<65	0.566	0.71 (0.23–2.25)	0.192	0.23 (0.03–2.07)
Smoking				
Yes		1.00 (Reference)		1.00 (Reference)
No	0.484	0.60 (0.14–2.51)	0.09	0.08 (0.00–1.47)
T stage				
1		1.00 (Reference)		1.00 (Reference)
2	0.266	2.50 (0.50–12.55)	0.07	8.64 (0.84–89.07)
3	0.823	1.20 (0.24–6.16)	0.671	0.59 (0.05–6.74)
4	0.987	1.02 (0.12–8.83)	0.772	0.65 (0.04–11.91)
N stage				
0		1.00 (Reference)		1.00 (Reference)
1	0.711	0.65 (0.07–6.35)	0.937	1.11 (0.08–15.01)
2	0.228	0.35 (0.06–1.93)	0.41	0.42 (0.05–3.35)

*Abbreviations*: *OR* Odds ratio, *CI* Confidence interval, *Ref* Reference. P values < 0.05 are shown in bold

**Fig. 2:**
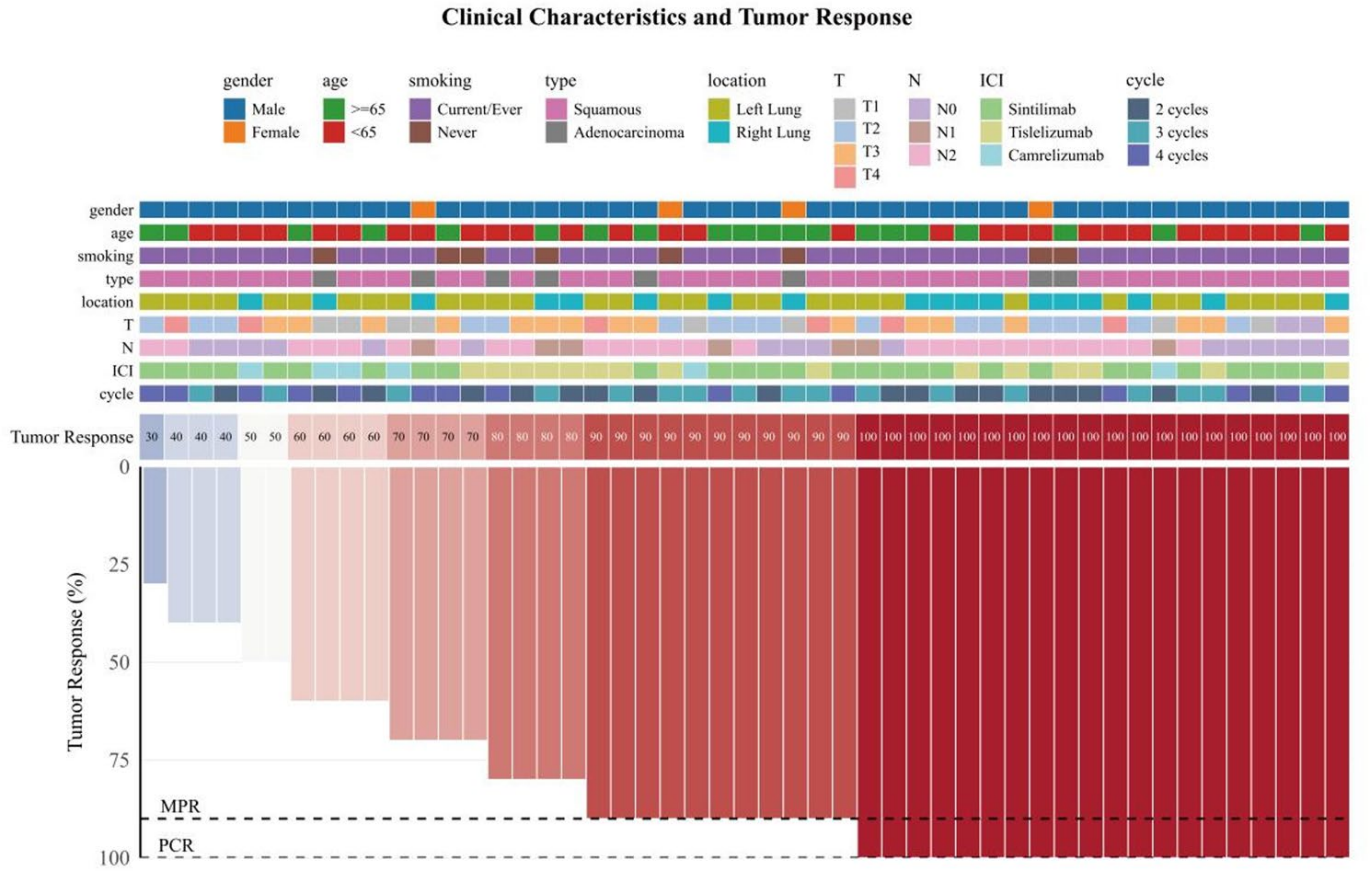
Waterfall plot of pathological response: Group A

**Fig. 3:**
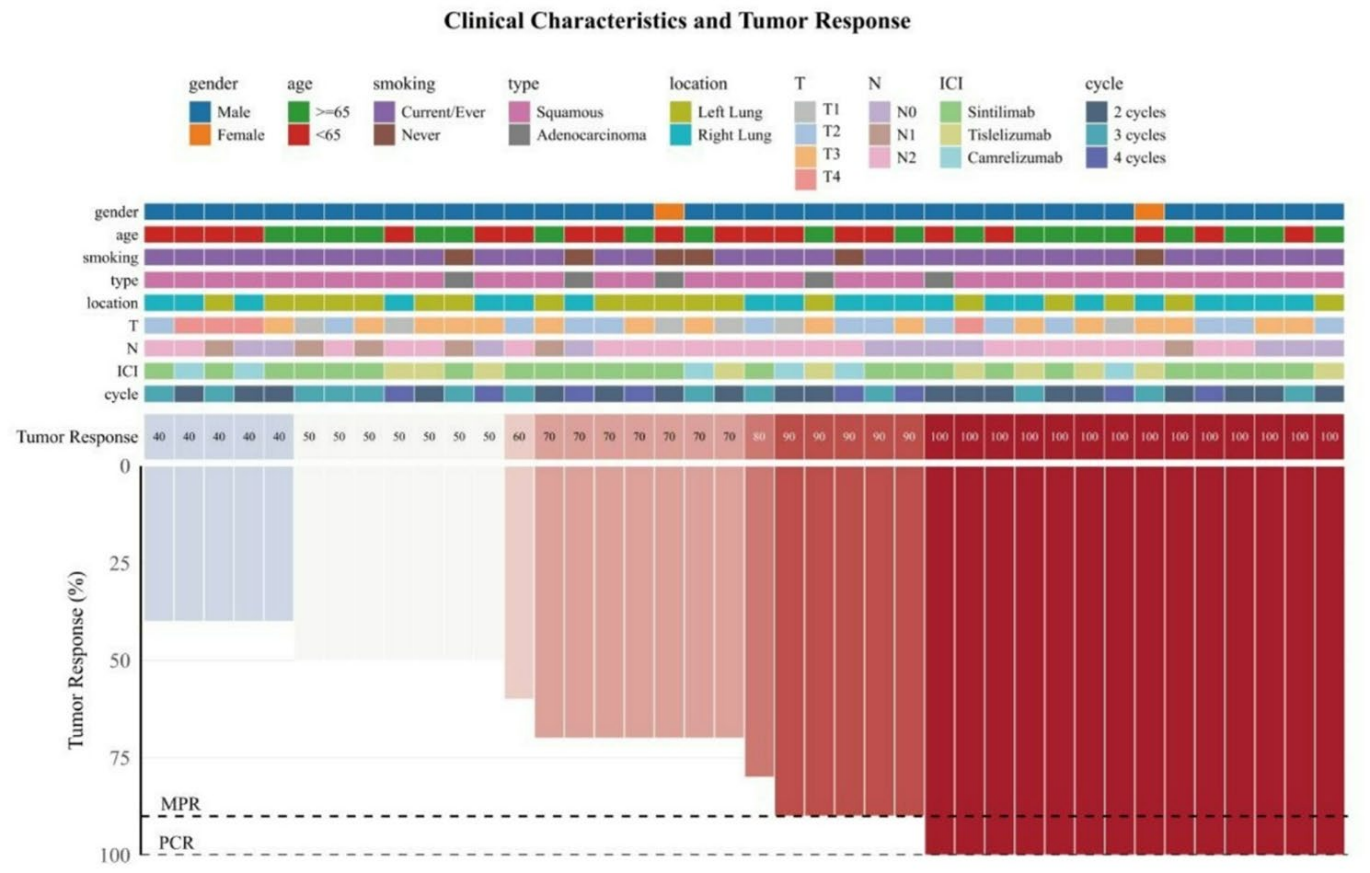
Waterfall plot of pathological response: Group B

Waterfall plots (Figs. 2 and 3) illustrate the depth of pathological regression in the primary tumor for each patient. The dashed line indicates the MPR threshold (≤ 10% residual viable tumor).

#### Sensitivity analysis

The E-value for the point estimate of the univariate OR (2.33) was 4.09, and the lower 95% CI limit corresponded to an E-value of 1.34. For the multivariable OR (4.78), the point estimate E-value was 9.03 and the lower 95% CI limit E-value was 1.79. These values exceed the typical range of common epidemiological confounders, suggesting that the observed association may be relatively robust to unmeasured confounding. Nevertheless, given the retrospective study design, the possibility of residual confounding remains.

#### ORR

After matching, ORR was significantly higher in Group A (45/62, 72.58%) than in Group B (32/62, 51.65%) (OR = 2.62, 95%CI 1.16–5.93, *P* = 0.020), consistent with the pathological response findings.

#### TRAEs

Among the 124 matched patients, 40 (32.30%) experienced grade ≥ 3 TRAEs. The incidence was numerically higher in Group A (22/62, 35.48%) than in Group B (18/62, 29.03%), but the difference was not statistically significant (OR = 1.27, 95%CI 0.64–2.49, *P* = 0.494). The most common grade ≥ 3 TRAEs in both groups were myelosuppression (Table 7). Severe adverse events (death/ICU admission) occurred in 4 patients (6.45%) in Group A and 1 patient (1.61%) in Group B.

**Table 7:** Grade ≥ 3 treatment-related adverse events

Adverse event	Group A (*n* = 22)	Group B (*n* = 18)
Leukopenia	8	5
Thrombocytopenia	2	1
Anemia	2	3
Nausea/vomiting	2	2
Liver dysfunction	2	1
Pneumonia	1	2
Thyroid dysfunction	2	1
Electrolyte imbalance	3	2
Myocarditis	0	1

## Discussion

This study is the first to systematically evaluate the impact of chemotherapy‑first versus immunotherapy‑first sequencing in neoadjuvant immunochemotherapy for stage IIA–IIIB NSCLC. After propensity score matching to minimize confounding, we found that the chemotherapy‑first sequence (Group A) significantly improved postoperative pathological response (50% vs. 30.64%; multivariable OR = 4.78, *P* = 0.023) and ORR (72.58% vs. 51.65%, *P* = 0.020) compared with the immunotherapy‑first sequence (Group B). These findings generate the hypothesis that the chemotherapy-first sequence may improve pathological response, providing a rationale for prospective studies to validate the optimal administration sequence in neoadjuvant immunochemotherapy for NSCLC.

Accumulating evidence confirms that pCR and MPR are robust surrogate endpoints for long‑term survival in NSCLC patients receiving neoadjuvant immunochemotherapy [30–33]. A meta‑analysis by Waser et al. demonstrated strong associations of pCR and MPR with EFS and overall survival [30]. Similarly, Tagliamento et al. reported R² values of 0.82 (pCR) and 0.81 (MPR) for 2‑year EFS [31]. These data support the relevance of our primary endpoint and imply that the observed improvement in pathological response may translate into survival benefits.

The biological rationale for sequence‑dependent efficacy lies in the temporal interplay between chemotherapy‑induced immunogenic cell death (ICD) and subsequent immune activation [34]. Platinum‑based and taxane chemotherapies can induce ICD, characterized by the release of damage‑associated molecular patterns (DAMPs) such as calreticulin, adenosine triphosphate (ATP), and high mobility group box 1 protein (HMGB1), which promote dendritic cell maturation and antigen presentation, thereby enhancing the tumor microenvironment for immune checkpoint inhibition [31–34]. ICD is not an instantaneous event; calreticulin exposure occurs within hours, ATP release persists for hours to days, and HMGB1 release is a late apoptotic event [35–37]. This temporal window suggests that there is an optimal interval between chemotherapy and ICI administration. The tumor immune microenvironment is not static during neoadjuvant therapy. Key immune‑related markers such as PD‑L1 and V-domain Ig suppressor of T-cell activation can be dynamically modulated by treatment, as demonstrated by Lv et al. in a study of patients with NSCLC receiving neoadjuvant chemotherapy or chemoradiotherapy [38]. In esophageal squamous cell carcinoma, Xing et al. found that administering toripalimab 48 h after chemotherapy yielded a markedly higher pCR rate than concurrent administration (36.4% vs. 7.7%), supporting the concept of a delayed‑immunotherapy window [19]. In our study, although the exact interval was not experimentally controlled, the overnight interval between chemotherapy and immunotherapy in Group A may have allowed initial ICD events to occur before ICI exposure, thereby maximizing synergy. Liang et al. published an exploratory study in which 38 patients with stage IIA-IIIA NSCLC were treated with a reduced-dose chemotherapy on day 1 combined with delayed immunotherapy on day 5 regimen. The results showed that the MPR rate was 47.4%, the pCR rate was 31.6%, and the ORR was 73.7%. Only one grade 3 adverse event occurred. This study suggests that chemotherapy followed by immunotherapy with an appropriate delay in the administration of immunotherapy may be a promising strategy worthy of further exploration [39].

Conversely, administering immunotherapy before chemotherapy (Group B) might expose activated effector T cells to subsequent cytotoxic chemotherapy, potentially attenuating the immune response. Zhu et al. showed in murine models that chemotherapy‑first sequences were superior, and that immunotherapy‑first schedules led to reduced intratumoral effector T cells [20]. This “immune‑depletion” hypothesis aligns with the lower pathological response we observed in Group B.

### Exploratory histological observation

**Fig. 4:**
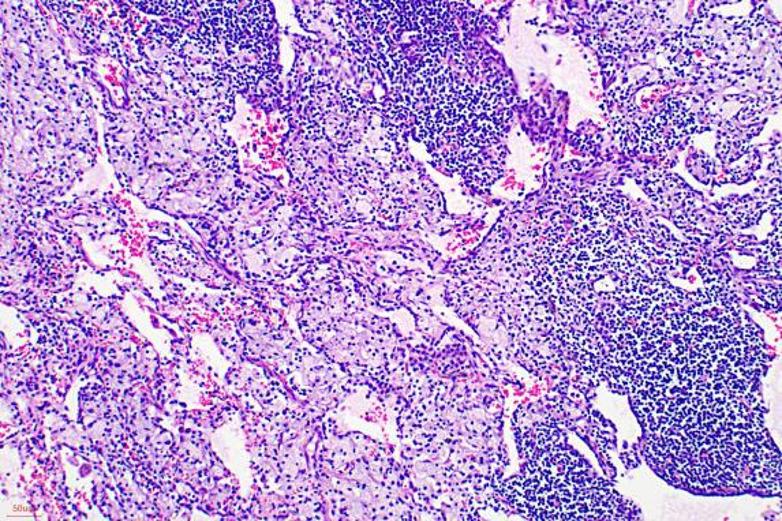
A1: Abundant foam cells and lymphocytic infiltration, occasional macrophages in alveolar spaces

**Fig. 5:**
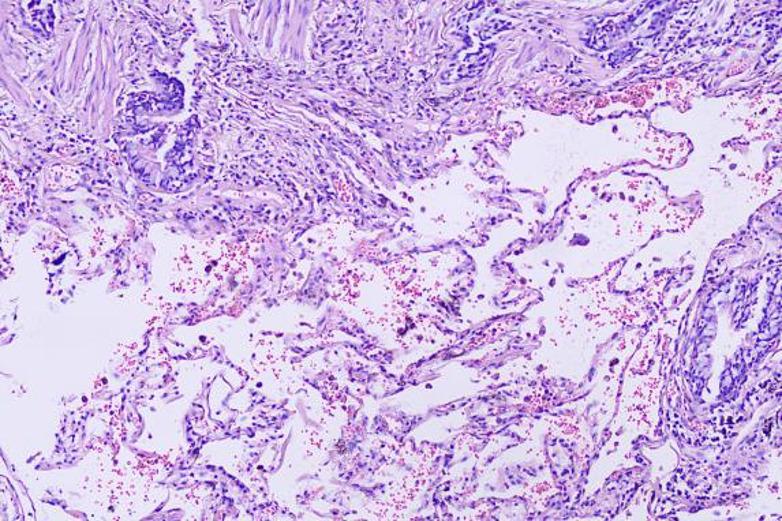
B1: Focal fibrous tissue hyperplasia, hyaline change, focal lymphocytic infiltration

**Fig. 6:**
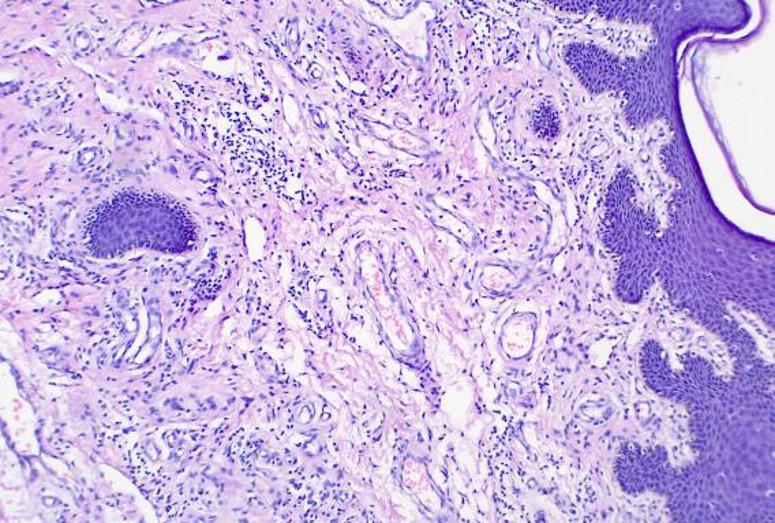
A2: Focal bronchial epithelial squamous metaplasia, sparse lymphocytes

**Fig. 7:**
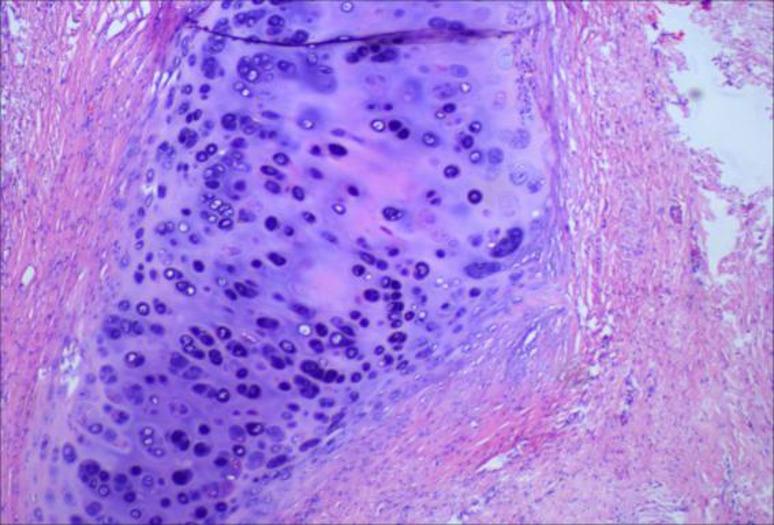
B2: Focal organizing pneumonia‑like change, scattered lymphocytes

**Fig. 8:**
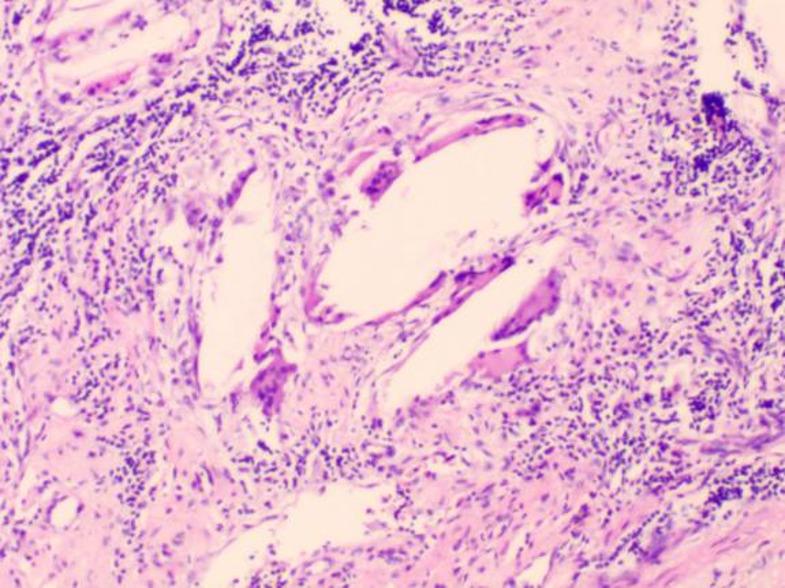
A3: Focal fibrous tissue proliferation, scattered lymphocytes, multinucleated giant cells, cholesterol clefts

**Fig. 9:**
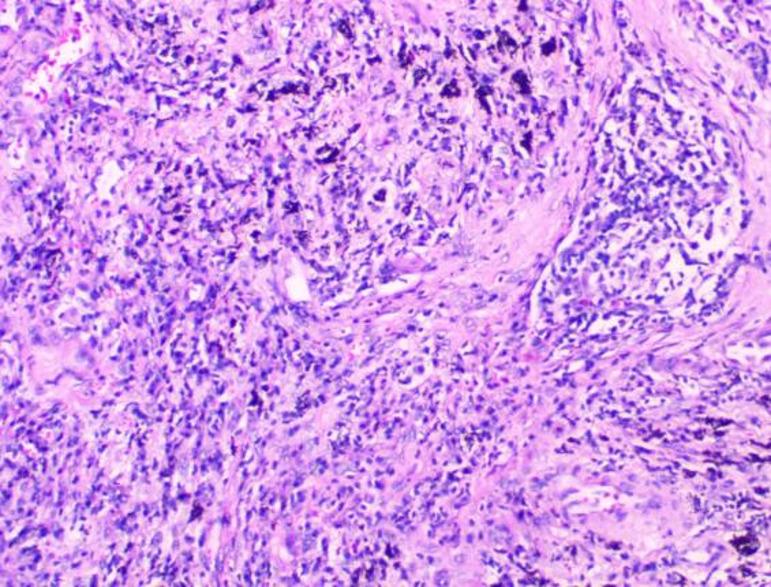
B3: Multifocal fibrotic change, focal bronchial epithelial squamous metaplasia, scattered lymphocytes

To initially explore potential histological differences of two groups, we randomly selected three pairs of specimens of patients who achieved pCR for hematoxylin‑eosin (HE) staining (six patients in total, Figs. 4, 5, 6, 7, 8 and 9). In these three pairs, patients in Group A (A1, A2, A3) appeared to show more pronounced lymphocytic infiltration, foam cell aggregation and cholesterol clefts, which may reflect a more active immune response, compared with patients in Group B (B1, B2, B3). These observations are consistent with the hypothesis that the chemotherapy‑first sequence may be associated with a more immunoreactive tumor microenvironment. Foam cells represent macrophages that have engulfed apoptotic tumor cells, reflecting active efferocytosis and antigen clearance, which are integral to ICD‑driven immunity [40]. Lymphocyte infiltration is a hallmark of ongoing immune responses [41]. These observations are preliminary and hypothesis‑generating. Given the very small sample size, these findings do not provide definitive mechanistic evidence. Consequently, further studies with larger cohorts and quantitative immune profiling are needed to further investigate these observations and elucidate the underlying mechanisms.

Several other studies have been exploring sequence‑related questions in various tumor types. In melanoma, the NeoTrio trial found that concurrent targeted therapy and PD‑1 blockade yielded the highest pathological response, while PD‑1 monotherapy produced more durable responses [42, 43]. In NSCLC, our results complement those of CheckMate 816 and other trials by highlighting the importance of sequence even within the established immunochemotherapy framework [12]. Importantly, the optimal sequence may be tumor‑specific, reflecting differences in immunogenicity, chemotherapy immunomodulatory effects, and ICI mechanisms.

To minimize bias inherent in retrospective analyses, we employed rigorous statistical methods. PSM balanced observed covariates, and E‑value analysis suggested that the observed association may be relatively robust to unmeasured confounding [29]. The E‑value for the multivariable OR was 9.03, meaning an unmeasured confounder would need to be associated with both treatment and outcome by an OR of > 9 to fully explain the observed effect. However, it is important to note that the E‑value does not completely eliminate the possibility of residual confounding. It only quantifies the strength that an unmeasured confounder would need to have. Given the retrospective design and non‑randomized treatment allocation, unmeasured confounding remains a potential concern.

The safety profile of the chemotherapy‑first sequence was acceptable. Grade ≥ 3 TRAEs were similar between groups, and although operative time was slightly longer in Group A, no significant differences were observed in the proportion of video‑assisted thoracoscopic surgery, unplanned conversions to thoracotomy, blood loss, postoperative complications, or length of hospital stay. The longer operative time may reflect increased tissue inflammation or fibrosis induced by robust immune activation [44]. The R0 resection rate in Group A (95.9%) was numerically higher than that in Group B (87.5%), but this difference did not reach the statistical significance level (*P* = 0.236). Although the R0 resection rate in Group B was comparable to that reported in CheckMate 816 (83%), it seems to indicate that patients with poor tumor response may have a higher risk of incomplete resection [12]. Nevertheless, given the risk of post‑treatment selection bias, these perioperative findings should be interpreted with caution and considered descriptive.

Several limitations should be acknowledged. First, this is a single‑center retrospective study. PSM can only balance measured covariates, but unmeasured factors may still have influenced treatment assignment and outcomes. Such factors include physician preference in sequence selection, subtle differences in patient fitness, tumor burden beyond TNM stage, scheduling logistics in clinical practice, and perioperative intent. The E‑value quantifies the strength of unmeasured confounders, but it does not prove that such confounding is absent. Therefore, our findings should be interpreted as hypothesis‑generating, and causal inference cannot be drawn from this study. Second, the multivariable analysis after propensity score matching was based on a modest sample size (62 matched pairs). While we adjusted for variables with residual imbalance (SMD > 0.1) to reduce confounding, this may have contributed to model instability and the wide confidence intervals observed (OR 4.78, 95%CI 1.24–18.46). Therefore, these estimates should be interpreted with caution. Consequently, larger prospective studies are needed to confirm the findings with greater precision. Third, long‑term survival data are not yet available; although pCR and MPR are validated surrogates, they do not fully replace direct assessment of EFS or OS. We are continuing follow‑up to provide these data in the future. Fourth, the mechanism explored via histology is preliminary and qualitative; larger‑scale studies with multiplex immunofluorescence or single‑cell sequencing are needed. Fifth, the optimal interval between chemotherapy and immunotherapy remains undefined; our study used a consistent overnight interval, but intervals of 24 h, 48 h, or longer may yield different results. Sixth, PD-L1 expression was not uniformly assessed, and other potential molecular biomarkers (e.g., KRAS, BRAF, MET) were not routinely tested due to the retrospective study period. Therefore, we could not evaluate their influence on treatment sequence outcomes. This should be addressed in future prospective studies. Finally, three different PD‑1 inhibitors (sintilimab, tislelizumab, and camrelizumab) were included in this study. Although their distribution was balanced between groups after propensity score matching, subtle differences in pharmacokinetics and PD‑1 occupancy may have influenced outcomes. Importantly, these agents are predominantly used in China and other Asian countries, which may limit the generalizability of our findings to regions where other PD‑1/PD‑L1 inhibitors are more commonly used. Furthermore, although platinum has been proven to be balanced, given that cisplatin and carboplatin may have different immunomodulatory properties, the effects of their interaction with different immunotherapeutic drugs remain unclear. Due to the relatively small sample size after matching, further stratification would lead to insufficient statistical power and unstable results. Although our findings may still provide relevant insights for clinical practice beyond the specific drugs used, future studies incorporating a broader range of immune checkpoint inhibitors and different platinums are needed to validate the sequence effect across different therapeutic contexts.

## Conclusion

In patients with stage IIA–IIIB NSCLC, the within-cycle administration sequence of chemotherapy followed by immunotherapy was associated with a higher postoperative pathological remission rate. It is suggested that this dosing sequence may have clinical optimization value, but further validation through prospective studies is still needed.

### Clinical Practice Points

What is already known about this subject? Neoadjuvant immunochemotherapy is standard for resectable NSCLC, but the optimal sequence of chemotherapy and immunotherapy within a treatment cycle is not defined.

What are the new findings? In this retrospective cohort with consistent overnight intervals, the chemotherapy‑first sequence was associated with a higher pathological response rate compared with the immunotherapy‑first sequence, without a significant increase in severe adverse events or compromise of surgical outcomes.

How might it impact clinical practice in the foreseeable future? The observed association suggests that the chemotherapy-first sequence may be a possibly promising strategy, but prospective studies are needed to confirm these findings.

## Supplementary information

Supplementary Material 1.

## Data Availability

The datasets generated during and analysed during the current study are available in the the database of the Affiliated Hospital of North Sichuan Medical College repository. The datasets generated during and analysed during the current study are available from the corresponding author on reasonable request (Daiyuan Ma, mdylx@163.com).
