# Supplementary material for: The influence of the administration sequence of neoadjuvant immunotherapy combined with chemotherapy on the postoperative pathological response of stage IIA-IIIB non-small cell lung cancer
Source: BMC Cancer. 2026 Apr 22;26:710. doi: 10.1186/s12885-026-16028-9 (PMC13238016; doi:10.1186/s12885-026-16028-9)
Supplement: Supplementary file 1 — Supplementary Material 1. [file 12885_2026_16028_MOESM1_ESM.pdf]

川北医学院附属医院医学伦理委员会伦理审查批件

IRB Review Approval Notice

批件号 File Number:2025ER197-1

项目名称 Study Title	新辅助免疫联合化疗用药顺序对可切除NSCLC术后病理缓解的影响：一项回顾性对照研究			
项目来源 Project source	研究生毕业课题			
审查类别 Review type	初始审查 Conference Review ☑； 修订后审查 Revised Review □； 跟踪审查 Follow-up review□			
审查方式 Review way	会议审查 Conference Review□； 快速审查 Quick Review☑			
主要研究者 Principal investigator	马代远 张宇	职 称 Professional title	主任医师	
承担科室 Research department	肿瘤科	联系电话Phone No.	17713801898	
审查日期 Review date	2025 年4月11日	审查地点 Address	–	
审核内容 Reviewed items	研究方案版本号 Study protocol version number	v1.0	研究方案版本日期 Study protocol version date	2025年3月24日
	知情同意书版本号 Informed Consent Form version number	–	知情同意书版本日期 Date of informed consent form	–
	其他文件 Other specify	豁免知情同意书		
主审委员 Presiding judge	杨汉丰□ 李敬东□ 张全波□ 任亦星□ 林菁艳☑ 马代远□ 蒋莉☑<br>陈天武□ 侯令密□ 刘凤君□ 曾玉华 □ 赵 婧□ 杨 明□ 余进洪□<br>熊永福□ 岳荣川□ 郭 斌□ 魏雪梅□ 李阳友□ 刘 辉□ 周士逵□			
审查结果 Decision:	同意(Approved)☑； 作必要修改后同意(Conditional approved)□；<br>作必要修改后再审(Reviewde after revising)□； 不同意(Disapproved)□；<br>终止或暂停先前批准的实验(Temination/Suspension)□			
年度/定期跟踪审查频率 Regular tracking review frequency	该研究进行过程中将接受伦理委员会的年度/定期跟踪审查频率<br>3 个月（3months)□； 6 个月（6months)□； 12 个月（1year)☑； 不适用(NA)□			
主任委员签字/盖章 Commissioner signature	杨汉丰印		[illegible]理委[illegible]会盖章 IRB S[illegible]ature<br>川北医学院附属医院医学伦理委员会	
签发日期 Date	2025 年 4 月16日			

地址：四川省南充市顺庆区茂源南路 1 号　　邮编：637000　　电话：0817-2262124

备注：

请遵循我国相关法律、法规和规章（《涉及人的生物医学研究伦理审查办法》等）以及 WMA《赫尔辛基宣言》和 CIOMS《人体生物医学研究国际道德指南》、遵循伦理委员会批准的方案和知情同意书开展临床试验/研究，保护受试者的健康与权力。

试验/研究开始前，建议申请人完成临床试验/研究注册。

试验/研究过程中若变更主要研究者，对临床试验/研究方案、知情同意书、招募材料等的任何修改，请申请人提交修正案审查申请。

发生严重不良事件，请申请人及时提交严重不良事件报告；紧急报告之后，尽快提交详细的严重不良事件随访报告。

请按照伦理委员会规定的年度/定期跟踪审查频率审查，提交试验/研究进展报告；当出现任何可能显著影响试验/研究进行、或增加受试者风险的情况时，请申请人及时向伦理委员会提交书面报告。

试验/研究纳入了不符合纳入标准或符合排除标准的受试者，符合中止试验/研究规定而未让受试者退出试验/研究，给予错误治疗或剂量，给予方案禁止的合并用药等没有遵从方案开展试验/研究的情况；或可能对受试者的权益/健康、以及试验/研究的科学性造成不良影响等违背伦理原则与规范的情况，请申办者/监查员/研究者提交违背方案报告。

申请人暂停或提前终止临床试验/研究，请及时提交暂停/终止试验/研究报告。

完成临床试验/研究，请申请人提交结题报告。

本项临床试验/研究应当在批准之日起半年内实施，逾期未实施的，则自行废止。
